# Single-Cell Transcriptomics for Unlocking Personalized Cancer Immunotherapy: Toward Targeting the Origin of Tumor Development Immunogenicity

**DOI:** 10.3390/cancers15143615

**Published:** 2023-07-14

**Authors:** Saeed Khodayari, Hamid Khodayari, Elnaz Saeedi, Habibollah Mahmoodzadeh, Alireza Sadrkhah, Karim Nayernia

**Affiliations:** 1 International Center for Personalized Medicine (P7MEDICINE), Luise-Rainer-Str. 6-12, 40235 Düsseldorf, Germany; s.khodayari@khu.ac.ir (S.K.); h.khodayari@hotmail.com (H.K.); 2 Oxford Clinical Trials Research Unit, Centre for Statistics in Medicine, Nuffield Department of Orthopaedics, Rheumatology and Musculoskeletal Sciences (NDORMS), University of Oxford, Oxford OX3 7LD, UK; elnaz.saeedi@ndorms.ox.ac.uk; 3 Breast Disease Research Center, Tehran University of Medical Sciences, Tehran 1819613844, Iran; 4 Hospital Du Jura, 2800 Delémont, Switzerland; alireza.sadrkhah@h-ju.ch

**Keywords:** cancer immunotherapy, personalized medicine, tumor development, cancer progression, single-cell transcriptomics, origin of tumor development

## Abstract

**Simple Summary:**

This study explains how the application of single-cell transcriptomics can enhance personalized cancer immunotherapy. Tumors exhibit complex and heterogeneous characteristics that can impede the effectiveness of immunotherapy. We specifically present the “Origin of Tumor Development” (OTD), consisting of undifferentiated tumor cells, which contribute to tumor diversity and heterogeneity. Using single-cell transcriptomics, scientists can analyze the gene expression profiles of individual tumor cells to gain insight into tumorigenesis, progression, and immune evasion. This approach enables the identification of personalized biomarkers and targets, including immune checkpoints and tumor-infiltrating lymphocytes, tailored to each patient. We also discuss future directions, such as the development of analytical tools and databases, to maximize the potential for targeting the patient’s OTD cells and advance personalized cancer immunotherapy.

**Abstract:**

Cancer immunotherapy is a promising approach for treating malignancies through the activation of anti-tumor immunity. However, the effectiveness and safety of immunotherapy can be limited by tumor complexity and heterogeneity, caused by the diverse molecular and cellular features of tumors and their microenvironments. Undifferentiated tumor cell niches, which we refer to as the “Origin of Tumor Development” (OTD) cellular population, are believed to be the source of these variations and cellular heterogeneity. From our perspective, the existence of distinct features within the OTD is expected to play a significant role in shaping the unique tumor characteristics observed in each patient. Single-cell transcriptomics is a high-resolution and high-throughput technique that provides insights into the genetic signatures of individual tumor cells, revealing mechanisms of tumor development, progression, and immune evasion. In this review, we explain how single-cell transcriptomics can be used to develop personalized cancer immunotherapy by identifying potential biomarkers and targets specific to each patient, such as immune checkpoint and tumor-infiltrating lymphocyte function, for targeting the OTD. Furthermore, in addition to offering a possible workflow, we discuss the future directions of, and perspectives on, single-cell transcriptomics, such as the development of powerful analytical tools and databases, that will aid in unlocking personalized cancer immunotherapy through the targeting of the patient’s cellular OTD.

## 1. Introduction

Based on the latest data released by GLOBOCAN 2020, the worldwide incidence of cancer has surged to 19.3 million cases, with a recorded 10 million deaths attributed to the disease in the year 2020 alone. This significant increase highlights the urgent requirement for the implementation of innovative and efficacious cancer therapies [1]. Cancer immunotherapy, as a potent treatment, is based on the leveraging of the immune system to kill the malignant cells. At present, several types of cancer immunotherapies are under development, with the most notable being checkpoint inhibitor therapy, cancer vaccination, and immune cell therapy [2,3]. However, the absence of target markers for the presentation of malignant cells to the immune system represents a substantial barrier to the advancement of effective cancer immunotherapy. Therefore, the identification of targeted markers is an indispensable requirement for a practical immunotherapeutic intervention [4].

Tumor heterogeneity encompasses the variability of cancer cells, spanning from their morphologies and genotypes to their functions [5,6]. This feature arises from various sources, such as genomic instability, epigenetic changes, microenvironmental factors, and selective pressures [7]. Tumor heterogeneity presents a significant challenge for a “one-size-fits-all” treatment approach, since the distinct diversity and biology exhibited by each tumor can significantly impact the effectiveness of the intervention. Accordingly, personalized medicine approaches that account for the heterogeneity of individual patients’ tumors have now emerged as a practical option in cancer treatment [8]. Advanced technologies like high-throughput transcriptomics facilitate personalized medicine in determining the optimal therapeutic strategy based on the unique characteristics of each tumor. Single-cell RNA sequencing (scRNA-seq), as a highly accurate transcriptomics method, offers the potential to profile the gene expression of individual cells within a tumor and uncover their cellular heterogeneity [9]. This powerful method greatly facilitates the identification of prospective biomarkers specific to each tumor’s cellular population and potential therapeutic targets for personalized cancer treatment [9,10].

Observations have shown the existence of populations with stem phenotypes within tumors [11,12,13], which may be the source of tumor heterogeneity and progression. As a dynamic developmental process, these carcinogenic stem populations, in response to the tumor microenvironment, may serve as the source of other tumor-malignant cells. We named this population the “Origin of Tumor Development” (OTD) and inferred that the targeted repression of this carcinogenic niche of the tumor can impede disease progression. Therefore, scRNA-seq represents an effective tool for detecting the OTD of a patient’s tumor, uncovering the specific immunogenic markers associated with their OTD, and assessing the function of the patient’s immune system against the OTD. Accordingly, in this review, we discuss the OTD as the basis of tumor heterogeneity and progression, the potential of scRNA-seq for efficient precision cancer immunotherapy targeting the OTD, and the prospects of this approach.

## 2. Current Status of Cancer Immunotherapy and Cancer Personalized Immunotherapy

The field of cancer immunotherapy has witnessed great advancements in recent years, and checkpoint inhibitor therapy, cancer vaccination, and immune cell therapy have emerged as promising strategies for personalized cancer treatment [2]. Studies have shown that the level of tumor mutational burden (TMB) status, programmed death-ligand 1 (PD-L1) expression, and tumor-infiltrating lymphocytes (TILs) are strongly correlated with the effectiveness of checkpoint inhibitors in certain types of cancer. Now, we clearly understand that cancer cells can suppress the immune system’s ability to produce an effective anti-tumor response. The primary suppressors of the tumor immune system, such as cytotoxic T-lymphocyte antigen-4 (CTLA-4) and PD-L1 immune checkpoints, can suppress T-cell cytokine production and proliferation [2]. High-TMB tumors tend to respond better to immunotherapy due to the creation of more neoantigens. Examples include melanoma, lung, bladder, and head and neck cancers, which also benefit from PD-1 or PD-L1 immune checkpoint inhibitors [14]. However, prostate, pancreatic, and glioblastoma tumors have a low TMB and tend to be resistant to immunotherapy, suppressing the immune system through various mechanisms such as reduced immune checkpoint expression or immunosuppressive molecule expression [15,16]. Therefore, the detection of tumor-specific immunogenicity through personalized medicine technologies is necessary to achieve efficient immunotherapy.

Moreover, the molecular and transcriptomic profiling of tumors and immune cells can help to identify appropriate targets for combination therapies or novel checkpoint inhibitors. Cancer vaccination aims to present specific antigens or neoantigens from malignant tissues to immune system effector cells [2]. These vaccines can be generated from patient tumor cells, circulating tumor cells (CTCs), or synthetic antigens [17]. Non-cancer vaccines with immunogenic potential have also shown favorable antitumor responses in some cancers. For example, a meta-analysis of the Bacillus Calmette–Guérin (BCG) vaccine on patients with bladder cancer found that it “significantly reduces the risk of progression to muscle-invasive disease after transurethral resection” [18]. However, not all patients derive benefits from cancer vaccines; thus, identifying biomarkers to select optimal antigens and delivery methods for each patient is crucial. Detecting the tumor’s neoantigens, which have suitable targeted immunogenicity, is a practical option. Next-generation sequencing (NGS) technology for the profiling of tumor cells or CTC genome sequencing provides a powerful tool with which to obtain these neoantigens for personalized cancer vaccination [19]. Transcriptomic and proteomic analyses of tumors and immune cells can also support the discovery of antigens or immune modulators for personalized cancer vaccines.

Personalized medicine can boost the effectiveness of cancer immune cell therapy by targeting the patient’s specific tumor immunogenic markers. This involves infusing autologous or allogeneic immune cells, such as TILs, T-cells, NK cells, dendritic cells, and macrophages, to boost their anti-cancer activity. NK cell and T-cell transfer therapies are commonly used for cellular-based cancer immunotherapy (CCIT) [2]. The tailoring of the treatment based on the patient’s immune system and targeting of specific tumor markers can increase the effectiveness of NK cell and T-cell therapy [2]. However, genetically engineered immune cells can further enhance CCIT by overcoming the limitations of classic CCIT, such as MHC restriction and tumor evasion. Chimeric antigen receptor (CAR-T) cell therapy is a cutting-edge approach in oncology that can recognize and selectively target specific antigens expressed in tumor cells [20]. However, it can cause substantial toxicities, including cytokine release syndrome, neurotoxicity, and B cell aplasia. Therefore, according to the ASCO guideline for the “management of immune-related adverse events in patients treated with CAR-T cell therapy”, the careful monitoring and management of potential toxicities are essential to ensure the safety and effectiveness of this intervention [21,22]. Tumor heterogeneity [6], immunosuppressive microenvironments, and off-target toxicity are some of the obstacles that remain. Scientists suggest that targeted and genetically engineered immune cells can increase the performance of CCIT [21].

The 2021 article authored by our group presented the current protocols, feasibility, and benefits of using stem-cell-derived NK cells for cancer immunotherapy as a potential CCIT strategy [2]. Our concept suggests that although peripheral-blood- or umbilical-cord-blood-derived NK cells can recognize and eliminate tumor cells without prior sensitization, their clinical application is limited due to issues with availability, functionality, and persistence. Therefore, we propose that patent-derived stem cells, including induced pluripotent stem cells (iPSCs) and mesenchymal stem cells (MSCs), could serve as alternative sources for generating effective, personalized NK cells. This idea is supported by several preclinical and clinical studies that evaluated stem-cell-derived NK cells for various types of solid tumors, including melanoma, glioblastoma, ovarian cancer, and hepatocellular carcinoma. Hence, it can be concluded that stem-cell-derived immune cells have tremendous potential for personalized cancer immunotherapy [2].

## 3. Individual Origin of Tumor Development: Concepts and Facts

**Figure 1:**
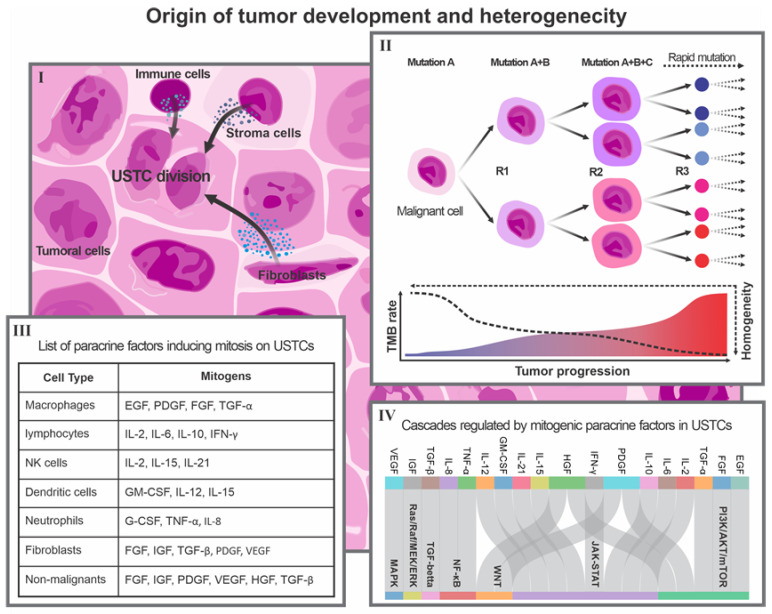
Description of the origin of tumor development as the basis of cancer heterogeneity. (**I**) Non-malignant tumor cells secrete factors into the tumor microenvironment that promote UTSC division. (**II**) The TMB rate increases due to the rapid division of tumor cells. This process generates different cell phenotypes and induces heterogeneity. (**III**) Table showing the main types of non-malignant tumor cells and their specific paracrine factors that can act as mitogens on USTCs. (**IV**) Sankey diagram showing the relationships between specific paracrine mitogens from non-malignant tumor cells and the main cascades regulating USTC biological processes.

The concept of OTD, or the tumor carcinogenic niche, revolves around the idea that two dynamic components, including undifferentiated stem tumor cells (USTCs) and the tumor microenvironment (TEM), contribute to the development of malignant tumors. The USTCs generate other tumor-associated cells that exhibit a malignant phenotype, while the TEM provides conditions conducive to tumor growth, development, and progression. The interaction between USTCs and the tumor microenvironment is critical in inducing USTC genesis, symmetrical proliferation, and eventual tumor progression (Figure 1).

USTCs are a population of undifferentiated malignant cells with stem-like phenotypes, capable of self-renewal and differentiation into various types of tumor cells. These phenotypes play a critical role in high-grade stem tumors, which are typically more aggressive and resistant to conventional therapies. The creation of USTCs is maintained through various mechanisms, including epithelial–mesenchymal transition (EMT), hypoxia, and dedifferentiation [23,24,25]. EMT is induced via multiple cascades, such as transforming growth factor Beta (TGF-β), wingless/integrated (Wnt), neurogenic locus notch homolog protein (Notch), nuclear factor kappa B (NF-κB), and hypoxia [26]. Moreover, hypoxia arises as a consequence of low oxygen levels in the tumor microenvironment, leading to the activation of hypoxia-inducible factor-1α (HIF-1α), which activates genes to promote CSC survival, proliferation, angiogenesis, and drug resistance, including octamer-binding transcription factor 4 (OCT4), nanog homeobox (NANOG), SRY-box transcription factor 2 (SOX2), vascular endothelial growth factor A (VEGFA), and ATP-binding cassette sub-family G member 2 (ABCG2) [27,28]. Dedifferentiation is also a process that can induce mature cells to revert to a stem-like state, which is mediated by various factors, including epidermal growth factor (EGF), sonic hedgehog (SHH), and DNA damage [25,29].

The TME is considered as an inseparable component of the tumor’s carcinogenic niche. The regulation of UTSCs is highly complex and involves several signaling pathways, such as Wnt, Notch, hedgehog, NF-κB, the Janus kinase–signal transducer and activator of transcription (JAK-STAT), the phosphatidylinositol-3 kinase/protein kinase B/mammalian target of rapamycin (PI3K/AKT/mTOR), and TGF-β (Figure 1). These pathways control the biological processes of various cell types, such as CSCs, including survival, proliferation, differentiation, plasticity, invasiveness, and drug resistance [30]. These facts indicate that the population of CSCs, as USTCs, are significantly influenced by the components of the TME, such as cytokines, growth factors, the extracellular matrix, hypoxia, and immune cells [31,32]. It has been demonstrated that stemness status can alter both the variation and expression levels of TME components [33]. These dynamic conditions, which are a key part of the OTD, play a critical role in maintaining stemness in high-grade tumors (Figure 1). 

Certain paracrine factors within the TME play a pivotal role in promoting the symmetric proliferation of USTCs, resulting in the parallel expansion of the OTD cellular population. Of particular interest are the paracrine factors with mitogenic functions, as they have the ability to stimulate the symmetric proliferation of USTCs, thereby promoting tumor expansion and growth. Notably, certain factors such as interleukin-6 (IL-6) and interleukin-8 (IL-8) are pro-inflammatory cytokines capable of activating the JAK-STAT and NF-κB pathways in USTCs, thereby enhancing their self-renewal and survival [34,35,36]. Similarly, growth factors like EGF and fibroblast growth factor (FGF) can bind to their respective receptors on USTCs, initiating the activation of the PI3K/AKT/mTOR and RAS/RAF/MEK/ERK pathways, which, in turn, promote USTC proliferation and differentiation [37]. Moreover, extracellular vesicles, including exosomes and microvesicles, contribute to USTCs’ behavior and fate by facilitating the transfer of proteins, lipids, and nucleic acids from donor cells to the USTCs, thereby influencing their stemness and plasticity [38]. Consequently, it becomes evident that the secretome and paracrine factors within the TME serve as critical regulators of USTCs’ behavior and fate. Furthermore, Figure 1(III) reveals the identification of additional paracrine factors with mitogenic functions in USTCs.

As previously mentioned, the microenvironment appears to play a crucial role in regulating the biological process of the OTD. Studies have shown that the implantation of malignant cells into the microenvironments of blastocysts or embryos can reprogram them into normal somatic cells or benign phenotypes, leading to the suppression of oncogene expression and activation of pluripotency transcription factors [39,40]. It has been observed that the direct implantation of melanoma cells into the cavity of mouse blastocysts led to the reprogramming of tumor cells, resulting in the development of chimeric species that showed no signs of tumor formation. This reprogramming was facilitated by the inhibition of the PI3K/AKT pathway and the up-regulation of the p53 pathway in melanoma cells [41]. The findings from these observations explain the pivotal significance of the microenvironment and its components in regulating the behavior, differentiation, and division of cancer cells.

Based on all the evidence explained, we believe that in the process from malignant cell creation to tumor formation and progression, we face a dynamic developmental process that forms the basis of the OTD. We propose that the OTD can be utilized to infer the best explanation of tumor heterogeneity. High-grade stem tumors are characterized by an elevated mutation rate, which is a significant contributor to the complexity and heterogeneity of malignant cells. The diverse genetic alterations found in USTCs, coupled with the influence of the TME, collectively contribute to this heterogeneity, ultimately influencing the phenotypes and behavior of tumor cells (Figure 1).

## 4. Single-Cell Transcriptomics for Detecting and Targeting the Immunogenicity of OTD

scRNA-seq is an advanced method that examines gene expression in individual cells, providing insights into cellular diversity and RNA patterns. It involves four main steps: isolating single cells, creating cDNA libraries, sequencing the libraries, and computationally analyzing the data [9,42]. Different methods exist for each step, with varying pros and cons (Table 1). The scRNA-seq offers a deeper understanding of cellular processes through, for example, identifying cell types, gene expression patterns, regulatory networks, and biological functions. The technique’s potential lies in its ability to uncover the complexity and dynamics of cellular systems in diverse biological contexts [10]. Table 1 summarizes various common types of scRNA-seq technologies, highlighting their capabilities, costs, and analytical methods.

As mentioned previously, the analysis of molecular diversity within heterogeneous tissues has been revolutionized using a powerful scRNA-seq method. This approach involves quantifying the expression level of the transcriptome at a single-cell resolution, providing researchers with the ability to distinguish between different cell types, phenotypes, states, and lineages, as well as their development and dynamics [43]. The scRNA-seq algorithm also allows for the identification of tissue lineages’ specific functions and behaviors through differential gene expression (DEGs) [43]. To improve the analysis of scRNA-seq data, numerous computational methods have been developed using mathematical and machine learning algorithms. These techniques aim to optimize scRNA-seq processing by addressing challenges related to high dimensionality, sparsity, noise, and batch effects [43,44]. 

Tumor heterogeneity and immune evasion pose significant challenges to the effectiveness of cancer immunotherapy. The population that complicates this issue significantly is the USTCs, which possess self-renewal and differentiation potential and constitute the primary component of the tumor’s overall tumor bulk. Identifying USTCs’ specific markers and their immunogenicity is critical for understanding tumor heterogeneity and developing predictive immunotherapy. However, detecting these markers is challenging due to the dynamic interconversion that occurs between the USTC phenotype and non-USTCs [45,46]. Conventional methods such as histological staining, microarray, and pooled-genome RNA-seq have limited resolution, as well as high noise, and cannot capture the diversity and dynamics of CSCs [47]. scRNA-seq can provide a comprehensive understanding of the molecular and functional characteristics of patent tumor undifferentiated stem phenotypes and their interactions with tumor immune cells [48]. It offers a unique tool for personalized immunotherapy targeting USTCs and overall tumor heterogeneity. Recent observations have shown that scRNA-seq can identify CSC-specific immunogenicity, including neoantigens and immune checkpoints, in various types of cancer [49]. Researchers have identified surface markers indicating the stem phenotypes within tumors. Nowadays, several global markers, such as CD44, CD24, CD90, CD133, and EPCAM, are used for detecting and isolating stem phenotypes in various cancers [50]. However, obtaining reliable and trustworthy levels of USTC markers from tumor-derived cells can be challenging due to several factors that may arise during library preparation. These factors include a low malignant cell yield when taking a tumor tissue biopsy from a margin, the loss of the cell population due to cell death during library preparation, the alteration of the cells’ transcriptional pattern during isolation, and even the destruction of isolated cell surface markers by protease enzymes during enzymatic cell isolation and culture. These confounding factors can affect the reliability and accuracy of the library preparation of tumor-derived cells and also reduce the scRNA-seq outcome in detecting an efferent marker for personalized immunotherapy [51].

Zhang et al. (2022) used a machine learning method to investigate the relationship between cancer stemness and immunotherapy response using scRNA-seq [52]. They proposed a novel stemness signature, called Stem.Sig, which was derived from scRNA-seq data obtained from patients undergoing treatment with immune checkpoint inhibitors (ICI). The study revealed a significant correlation between cancer stemness, as measured via CytoTRACE (https://cytotrace.stanford.edu, accessed on 5 June 2023), and ICI resistance in melanoma and basal cell carcinoma based on pan-cancer data. Stem.Sig demonstrated negative associations with anti-tumor immunity while showing positive associations with intra-tumoral heterogeneity and mutational burden. Remarkably, the machine learning model utilizing Stem.Sig outperformed other existing signatures in predicting ICI response across various cancer types. Furthermore, this research identified several potential therapeutic targets, including EMC3, BECN1, VPS35, PCBP2, VPS29, PSMF1, GCLC, KXD1, SPRR1B, PTMA, YBX1, CYP27B1, NACA, PPP1CA, TCEB2, PIGC, NR0B2, PEX13, SERF2, and ZBTB43, for stem tumors. These findings present promising avenues for effective immunotherapy for stem tumors [52]. These observations offer valuable insights into immune resistance mechanisms and have implications for the development of improved cancer treatment strategies.

The utilization of the liquid biopsy approach represents the optimal solution for addressing this challenge. Liquid biopsy is a practical method used to detect and isolate CTCs from a patient’s whole blood, which can provide critical information regarding tumor personalized features, development, and progression [53,54]. It has been reported that CTC populations in patients demonstrate the same heterogeneity as the tumors from which they originate [55]. Furthermore, since the emergence of the EMT theory, it has become evident that most CTCs exhibit a stem phenotype as they transition from an epithelial to a stem cell phenotype. The majority of the approved CTC isolation protocols utilize a gradient centrifugation method to purify CTCs from peripheral-blood-derived mononuclear cells (PBMCs). This approach does not induce substantial stress, thereby allowing the cells to retain their true immunogenic features following scRNA-seq [56]. 

**Figure 2:**
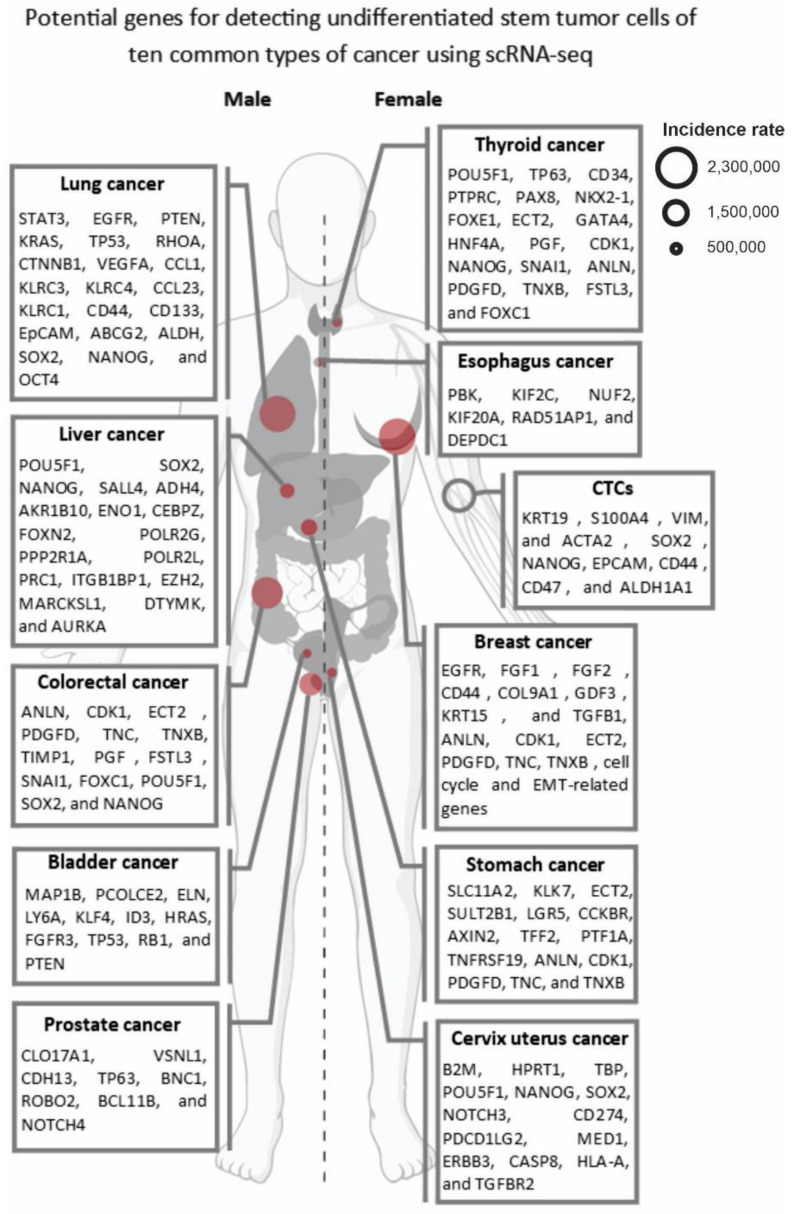
Potential genes that can be used to detect the USTCs of ten common types of cancers using scRNA-seq. The size of the red circles indicates the incidence rate of each cancer according to the latest, updated GLOBOCAN data (2020).

To perform personalized immunotherapy for UTSCs in the OTD, various mechanisms and markers can be targeted through scRNA-seq analysis. For instance, cancer/testis antigens (CTAs) like MAGE-A and NY-ESO-1 are expressed in many tumors and have decreased expression in normal tissues [57]. The MAGE-A genes are a group of CTAs encoded by the MAGEA gene family, consisting of 12 genes (MAGEA1–6, 8–12) located on the X chromosome, and expressed in various cancers [58]. NY-ESO-1 is another CTA encoded by the CTAG1B gene located on chromosome Xq28 and is widely detected in melanoma, lung, ovarian, breast, and prostate cancer, as well as in normal testis tissue [59]. A study conducted by Gordeeva et al. (2018) analyzed the co-expression landscape of 17 CTAs in 5450 tumors from 39 histologic types [60]. They observed that CTAs have a tendency to co-express in clusters, forming expression patterns characteristic of tumor subgroups. They also identified XAGE1B and GAGE10 as potential biomarkers for lung cancer and neuroendocrine tumors, respectively [60]. According to this information, detecting specific CTA expression in UTSCs through scRNA-seq could be an effective and practical method for personalized immunotherapy for the OTD, particularly in females. As part of our collective efforts, we are trying to identify potential CTA markers of breast cancer to facilitate personalized tumor immunotherapy. In 2012, we investigated the expression of CTGs Tsga10, TEX101, and ODF3 in patients with breast cancer [46]. Our study found that Tsga10 was expressed in 70% of the patients, while TEX101 and ODF3 were not expressed in any of the patients. Moreover, we observed that 14% of the patients had autoantibodies against Tsga10. Therefore, these findings suggest that Tsga10 may contribute to the proliferation and survival of breast cancer cells, and it could be a promising target for personalized breast cancer immunotherapy [61]. Additionally, a study that we published in *Cancer Research* in 2010 investigated the expression of CTG Piwil2 in breast CSCs. The study found that Piwil2 was highly expressed in breast CSCs, and its silencing suppressed the expression of the signal transducer and activator of transcription 3, a regulator of Bcl-XL and cyclin D1, leading to reduced cell proliferation and survival. These findings indicate that Piwil2 and its signaling pathways could be critical factors in the proliferation and survival of breast CSCs and used for the targeted therapy of breast UTSCs [62]. Overall, the scRNA-seq can aid in the identification and characterization of immunogenic markers on UTSCs and facilitate the development of personalized cancer immunotherapies that target these markers. Figure 2 summarizes the potential markers of USTCs for ten common cancer types.

## 5. Future Steps

**Figure 3:**
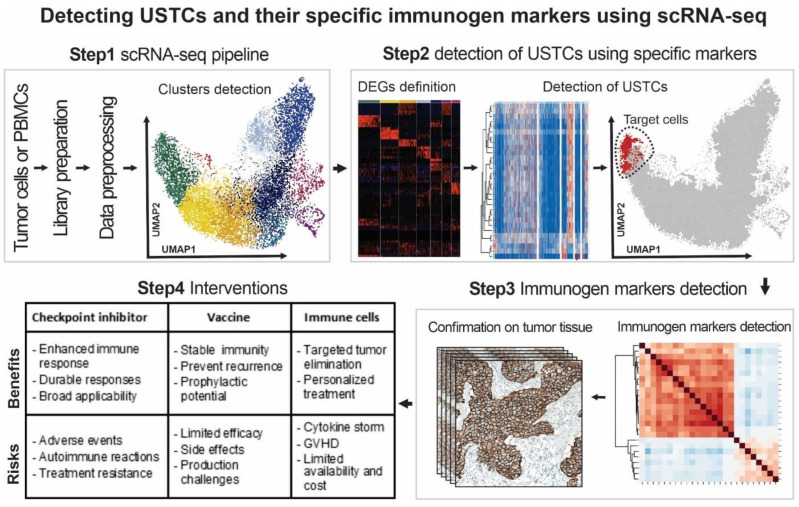
A potential workflow for detecting patients’ USTC immunogenic markers using scRNA-seq technology. This workflow consists of four main steps: Step 1: scRNA-seq pipeline for library preparation, data processing, and cluster detection of patients’ tumor-derived cells and/or PBMC. Step 2: detection of the USTCs using the markers. Step 3: discovery and validation of the detected USTC immunogenic markers in the tumor samples using different methods, such as immunohistochemistry (IHC) or Western blot. Step 4: selection of the most suitable and effective approaches for the personalized immunotherapy intervention of the patients.

**Figure 4:**
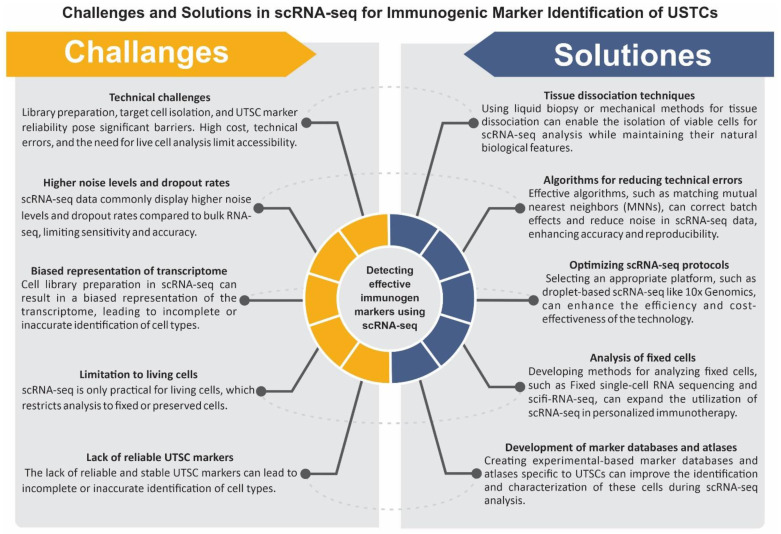
Challenges and solutions in using scRNA-seq technology to identify effective immunogenic markers of USTCs for OTD targeting.

In the previous chapter, we discussed some technical factors that can influence the quality of scRNA-seq outcomes in the detection of USTCs. In this chapter, we aim to provide a wide overview of the challenges associated with utilizing scRNA-seq technology for the personalized immunotherapy of the OTD. We present effective solutions to address these challenges and provide a practical workflow based on our experiences (Figure 3). Moreover, in Figure 4, the challenges and approaches in applying scRNA-seq technology to find potent immunogenic markers of USTCs for targeting the OTD are presented.

scRNA-seq technology, similar to other technologies, offers numerous benefits, but it is crucial to consider its limitations when interpreting results. Technical challenges related to library preparation, target cell isolation, and the reliability of UTSC-specific markers, along with the high cost of the technology, technical errors, and the fact that scRNA-seq is only applicable to living cells, pose significant barriers. To date, several scRNA-seq technologies have been presented, with varying abilities and improvements [63]. For instance, droplet-based scRNA-seq techniques such as 10× Genomics and Drop-seq have improved throughput and reduced the cost per cell [64]. Meanwhile, full-length transcript sequencing technologies such as Smart-seq2 and Smart-seq3 have enabled the sequencing of full-length transcripts, enhancing gene expression quantification and isoform identification [65]. Additionally, technologies such as 10× Genomics Visium and Spatial Transcriptomics have been developed for the analysis of transcriptome expression in its spatial context. The cost of operating scRNA-seq varies depending on the setup, with droplet-based scRNA-seq typically being less expensive per cell than full-length transcript sequencing technologies. As we determined, a combined run of four samples using 10× Genomics costs USD 6600, according to the pricing mentioned on the website (https://www.bumc.bu.edu/singlecell/pricing, accessed on 7 June 2023). This indicates that it is crucial to carefully select a suitable single-cell transcriptomics technology in personalized medicine that is cost-effective, accurate, and powerful. 

scRNA-seq data commonly display higher noise levels and dropout rates compared to bulk RNA-seq [66,67]. In bulk RNA-seq, the data only capture a small fraction of the cell’s mRNA expression. To address these limitations, novel library preparation techniques have been developed to reduce bias and improve the sensitivity of scRNA-seq analysis. For instance, batch effects are systematic differences in scRNA-seq data that arise from variations in experimental conditions, such as cell isolation methods, library preparation kits, sequencing platforms, and data analysis pipelines [68]. Noise is random variation in scRNA-seq data that results from biological factors, such as the cell cycle stage, cell size, and cell viability, or technical factors, such as amplification bias, dropout events, and sequencing errors [69]. Moreover, scRNA-seq still encounters several limitations that should be taken into account when interpreting results for personalized immunotherapy of cancer. One of the main technical challenges of scRNA-seq is cell library preparation, which can result in a biased representation of the transcriptome [68,69]. Moreover, scRNA-seq is only practical for living cells, which limits the analysis of cells that have been fixed or preserved. This also restricts the accessibility of the technology for almost all subjects. Additionally, the lack of reliable and stable UTSC markers can lead to the incomplete or inaccurate identification of cell types [70,71]. 

To overcome the challenges associated with scRNA-seq technology in the management of personalized immunotherapy for USTCs, several solutions can be considered. Using liquid biopsy or mechanical methods for tissue dissociation enables the isolation of viable cells for scRNA-seq analysis while maintaining their natural biological features. Tissue dissociation techniques employing mechanical methods involve the application of physical forces to disintegrate tissues into individual cells. Noteworthy mechanical approaches include manual mincing, homogenization, and the employment of microfluidic devices [72]. Manual mincing, characterized by the employment of scalpel or scissors, stands as a simplistic and cost-effective technique; however, it possesses limitations in terms of consistency and potential cell damage [72]. Homogenization, on the other hand, utilizes rotating blades or pistons to shear tissues but may generate heat and foam, thereby influencing cell viability. Microfluidic devices, comprising microchannels and hydrodynamic forces, serve to disrupt tissues, albeit at a relatively higher cost and necessitating specialized equipment [73]. Mechanical methods offer distinct advantages in facilitating prompt and efficient tissue dissociation for scRNA-seq analysis [73]. Moreover, this can overcome the limitations of using fixed or preserved cells, which do not qualify for scRNA-seq analysis due to RNA degradation [74]. On the other hand, the optimization of scRNA-seq protocols can significantly enhance the efficiency and cost-effectiveness of this technology, making it a more reliable and trustworthy single-cell transcriptomics method. This can be performed by selecting an appropriate single-cell transcriptomics platform that is cost-effective, accurate, and powerful, such as droplet-based scRNA-seq technologies like 10× Genomics, which has a high throughput and low cost per cell [75].

Batch effects and noise can affect the accuracy and reproducibility of scRNA-seq data. As previously explained, batch effects and noise are two sources of variation commonly observed in scRNA-seq data, arising from systematic differences in experimental conditions and random biological or technical factors, respectively [66,67,68,69,70]. Several methods and algorithms have been developed to mitigate the impact of batch effects and noise in scRNA-seq data. These methods aim to enhance the comparability and quality of scRNA-seq data across various samples [75,76]. For example, matching mutual nearest neighbors (MNNs) in the high-dimensional expression space is an effective and useful approach to correcting batch effects in scRNA-seq data. the MNNs are pairs of cells from different batches that exhibit similar gene expression profiles. By identifying and aligning MNNs across different batches, the MNN method can correct batch effects while preserving the biological format of the data. The MNN method is a highly accurate, scalable, and robust integration method that can be effectively utilized in personalized medicine approaches for cancer [77].

Furthermore, the development of experimental-based marker databases and atlases is essential for accurately identifying and characterizing UTSCs. These markers are genes specifically expressed or regulated in UTSCs, enabling differentiation from other cellular populations during scRNA-seq analysis (Figure 2 and Figure 3). Tumor stem cell marker databases and atlases provide a comprehensive collection of UTSC-specific markers to minimize incomplete or inaccurate identification. The Human Cell Atlas (HCA) is a global initiative that aims to map all human cells based on their molecular signatures, origins, and spatial locations. The development of UTSC marker databases utilizing the HCA algorithm can offer valuable insights into the distribution and diversity of these cells in different tissues and organs [68,78,79]. Although single-cell transcriptomics technology is primarily used for live cells, the development of methods for using fixed cells can help to extend scRNA-seq analysis to fixed cells in cancer personalized medicine applications. Fixed cells are cells that have been treated with chemical agents or physical methods to preserve their structure and transcriptome [80,81]. The fixed cells can be obtained from various sources and reserved for long periods without compromising their RNA quality. The development of new protocols for the isolation and preparation of fixed cells can expand the utilization of scRNA-seq analysis for personalized immunotherapy targeting patients’ OTD. For instance, some investigations have reported on the successful scRNA-seq analysis of fixed cells using methods such as fixed single-cell RNA sequencing [80] and single-cell combinatorial fluidic indexing RNA sequencing (scifi-RNA-seq) [81]. We believe that by implementing these solutions, scRNA-seq can become a powerful tool in the management of personalized immunotherapy for USTCs.

## 6. Conclusions

In this review, we aim to provide a brief explanation of the OTD concept as the main origin of tumor progression and heterogeneity using our observations and expe-rience in the field of cancer biology and personalized medicine. Additionally, we high-light the powerful application of scRNA-seq, which allows for the detection and dis-tinction of malignant undifferentiated stem phenotypes from other cell populations, providing insights into the cells’ specific immunobiology. While this approach has the potential to improve cancer immunotherapy performance, there are still challenges that need to be addressed, such as optimizing the process of cell isolation and prepara-tion protocols, expanding the availability of scRNA-seq facilities, reducing costs, and creating specific databases and atlases for USTCs’ markers to enable the globalization of this approach.

## Figures and Tables

**Table 1:** A description of the key parameters and applications of various scRNA-seq methods.

Method	Technology Name	Minimum Cells	Developer Company	Advantages	Disadvantages	Cost	Library Preparation Time	Sequencing Depth	Applications	Platforms for Analysis
Droplet-based	Drop-seq	1000	Macosko Lab	High throughput, low cost per cell, UMI-based quantification	Low coverage, limited information on isoforms, SNPs and VDJ rearrangements, cell doublets may occur	USD 0.06–0.2 per cell	1–2 days	0.1–0.5 million reads per cell	Cell type identification, gene expression profiling, trajectory inference	Seurat, Monocle, Scanpy
	inDrop	1000	Klein Lab and Shalek Lab	High throughput, low cost per cell, UMI-based quantification, flexible barcode design	Low coverage, limited information on isoforms, SNPs and VDJ rearrangements, cell doublets may occur	USD 0.06–0.2 per cell	1–2 days	0.1–0.5 million reads per cell	Cell type identification, gene expression profiling, trajectory inference	Seurat, Monocle, Scanpy
	Chromium 10×	500–10,000	10× Genomics	High throughput, low cost per cell, UMI-based quantification, multiple applications (e.g., immune profiling, spatial transcriptomics)	Low coverage, limited information on isoforms, SNPs and VDJ rearrangements, cell doublets may occur	USD 0.55–1.1 per cell	1–2 days	0.5–2 million reads per cell	Cell type identification, gene expression profiling, trajectory inference, immune repertoire analysis, spatial transcriptomics	Cell Ranger, Seurat, Monocle, Scanpy
Full-length	Smart-seq2 (SS2)	1–96	Picelli Lab and Sandberg Lab	High coverage, detection of isoforms, SNPs and VDJ rearrangements, low technical noise	Low throughput, high cost per cell, no UMI-based quantification	USD 35–70 per cell	2–3 days	5–20 million reads per cell	Isoform detection and quantification, SNP calling and phasing, VDJ rearrangement analysis	Cufflinks, DESeq2, edgeR
	Smart-seq3 (SS3)	1–96	Sandberg Lab and Linnarsson Lab	High coverage, detection of isoforms, SNPs and VDJ rearrangements, low technical noise, UMI-based quantification	Low throughput, high cost per cell, requires fine-tuning to balance internal and UMI-containing reads	USD 35–70 per cell (estimated)	2–3 days	5–20 million reads per cell	Isoform detection and quantification, SNP calling and phasing, VDJ rearrangement analysis	Cufflinks, DESeq2, edgeR
	FLASH-seq (FS)	1–96	Picelli Lab	High coverage, detection of isoforms, SNPs and VDJ rearrangements, low technical noise, UMI-based quantification with reduced strand-invasion artifacts, fast and simple protocol	Low throughput, high cost per cell	USD 35–70 per cell (estimated)	<4.5 h	5–20 million reads per cell	Isoform detection and quantification, SNP calling and phasing, VDJ rearrangement analysis	Cufflinks, DESeq2, edgeR

Terms: **SNPs**: Single-Nucleotide Polymorphisms; **UMI**: Unique Molecular Identifier; **VDJ**: rearrangement analysis, analyzing gene rearrangements in immune cells.
